# Application of Failure Criteria on Plywood under Bending

**DOI:** 10.3390/polym13244449

**Published:** 2021-12-18

**Authors:** Miran Merhar

**Affiliations:** Department of Wood Science and Technology, Biotechnical Faculty, University of Ljubljana, Jamnikarjeva 101, 1000 Ljubljana, Slovenia; Miran.Merhar@bf.uni-lj.si; Tel.: +386-1-320-36-29

**Keywords:** max stress, Tsai-Wu, Tsai-Hill, Puck, Hoffman, Hashin, failure criteria, beech, finite element modelling, composites

## Abstract

In composite materials, the use of failure criteria is necessary to determine the failure forces. Various failure criteria are known, from the simplest ones that compare individual stresses with the corresponding strength, to more complex ones that take into account the sign and direction of the stress, as well as mutual interactions of the acting stresses. This study investigates the application of the maximum stress, Tsai-Hill, Tsai-Wu, Puck, Hoffman and Hashin criteria to beech plywood made from a series of plies of differently oriented beech veneers. Specimens were cut from the manufactured boards at various angles and loaded by bending to failure. The mechanical properties of the beech veneer were also determined. The specimens were modelled using the finite element method with a composite modulus and considering the different failure criteria where the failure forces were calculated and compared with the measured values. It was found that the calculated forces based on all failure criteria were lower than those measured experimentally. The forces determined using the maximum stress criterion showed the best agreement between the calculated and measured forces.

## 1. Introduction

The use of wood as a sustainable material is increasing. Wood has anisotropic mechanical properties [1], but when the principal axes coincide with the orientation of the tissue, wood can be considered orthotropic and, in certain cases, transversely isotropic. One such example is wood composite, including plywood. The ratios of mechanical properties between the longitudinal and transverse directions of wood are between 10 and 1 [1], which are greatly reduced in plywood where the veneer sheets are glued together at different angles [2,3].

Plywood is a very common and long-used building material. It can be made from veneers of different tree species, which affects the physical and mechanical properties of plywood [4,5,6,7,8]. Important factors influencing the properties of plywood include design features such as the number, thickness and orientation of individual layers and the technological process of panel production [9,10,11,12,13,14,15].

Since different combinations of the listed factors can be used to obtain different properties, many authors have already dealt with the determination of the properties. Some have determined the modulus of elasticity [4,16,17], others the shear modulus [18,19], while still others have measured the strength of already manufactured plywood.

However, since plywood is composed of individual layers of veneer, it is important to know the mechanical properties in all directions of the wood, as the mechanical properties can vary greatly between longitudinal and transverse direction. In addition, the strength of wood also varies when it is subjected to tension or compression. Therefore, even for uniaxial stress conditions, a distinction must be made between compressive and tensile loads [1]. For a multi-axial stress state, in addition to the distinction between compression and tension, the strength in different directions must also be considered, for both normal and shear strength.

The relationships between the actual stresses and the corresponding strengths, and the consideration of whether or not the specimen will fail under specific load, are governed by various failure criteria. One of the most commonly used criteria are max stress [20], Tsai-Hill [21], Hoffman [22], Hashin [22], Tsai-Wu [23] and Puck [24,25], shown in Table 1. While the Tsai-Hill, Hoffman and Tsai-Wu criteria can only be used to determine the failure load or to determine whether the specimen will fail at a particular combination of stresses, the max stress, Puck and Hashin criteria can also be used to determine the failure mode, i.e., fibre or inter-fibre failure.

Most researchers have studied the application of failure criteria to unidirectional (UD) composites [26,27,28,29,30,31,32,33,34]. In these cases, the criteria predict the failure loads more or less satisfactorily. In a UD composite, the failure of the matrix usually implies the failure of the entire specimen. In a composite material, such as a plywood, where the individual layers are arranged at different angles, the failure of a particular layer in the transverse direction (matrix) does not necessarily imply the failure of the entire specimen. Thus, an adjacent layer whose fibres are oriented at a certain angle to the preceding layer may arrest the progression of the failure of the matrix, which in some cases can also carry the load of the preceding layer broken by the matrix.

Furthermore, the described criteria have already been used in the calculation of failure forces in oriented wood specimens loaded with different combinations of normal and shear stresses [35,36,37]. In doing so, the researchers analytically determined the normal and shear stresses as the function of grain direction and loading force and then inserted them into various criteria. However, as far as the author is aware, the application of these criteria in determining the failure forces of wood composites such as plywood, where the individual veneer layers are oriented differently, cannot be found anywhere in the literature. Thus, to determine the failure force of the entire plywood specimen, one must know the magnitude and direction of the stress in individual veneer layers and then determine the failure load for each individual layer. This is only possible by using the finite element method to get the result with the required accuracy. 

The aim of this study is therefore to investigate the applicability or accuracy of various failure criteria in determining the failure forces of plywood loaded in bending. Due to the structure of the panel with differently oriented veneers, each ply exhibits a different biaxial stress state, which makes the application of classical bending mechanics very difficult. From this point of view, the use of failure criteria in combination with the finite element method proves to be the most suitable when it comes to determining the failure force in each layer of the panel. These forces are compared with the experimentally determined values, and verified the application of failure criteria’s in wood composites such as plywood.

## 2. Materials and Methods

### 2.1. Plywood Processing

Peeled beech (*Fagus sylvatica*) veneer of 600 mm × 600 mm with tangential texture and nominal thickness of 1.5 mm was used to produce the plywood. The veneer was free from visual defects with homogeneous texture and was obtained from a single log with uniform growth of annual rings. The veneer was conditioned in the laboratory at a constant temperature of 22 °C and relative air humidity of 45%. After conditioning, the veneer had an average moisture content of 6.7%.

**Figure 1:**
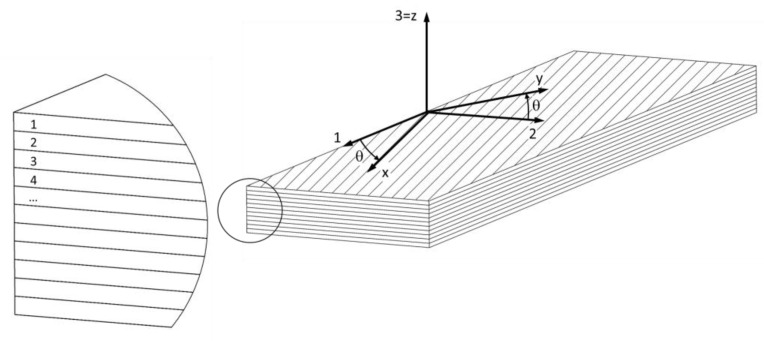
Plywood with specimen coordinate system (1-2-3) and principal material coordinate system (x-y-z).

The veneers were used to produce 11-, 7- and 3- ply plywood panels with the veneer orientations shown in Table 2 and Figure 1. The panels marked 11E were manufactured with the same orientations of all veneer plies. They were used to fabricate test specimens to determine the mechanical properties in the principle directions of the wood tissue.

Meldur H97 melamine-urea-formaldehyde (MUF) adhesive was used, provided by Melamin d.d. (Kočevje, Slovenia). According to the manufacturer, MUF adhesive consisted of 62% ± 2% dry content, viscosity (as per SIST EN ISO 2431 (2019) ϕ4, 20 °C) was 80 s to 200 s and consisted of maximum 0.5% free formaldehyde. To the adhesive 1% NH_4_Cl catalyst and 5% filler (rye flour) were added to increase viscosity. The mixture was then stirred for 15 min until a homogeneous mixture was obtained.

The adhesive application to the individual veneer layers was 180 g/m^2^. The pressing of the board was 1.6 MPa, the temperature 130 °C and the pressing time was 13 min, 10 min and 7 min for 11-, 7- and 3- layer plywood, respectively. After pressing, the boards were stacked, weighed and conditioned for 1 week.

After conditioning, the boards were cut to the size of 550 mm × 550 mm, and their thickness was measured. The seven-layer board was 9.9 mm thick, while the 11-layer board was 15.6 mm thick, corresponding to 1.42 mm per layer. 

Mechanical properties of plywood building material, i.e., veneer sheets in longitudinal, radial and tangential directions were determined. Tensile strength was determined on specimens of single veneers and compressive strength, shear strength, modulus of elasticity and shear modulus on specimens cut from 11E plywood.

### 2.2. Determination of Principal Mechanical Properties of Plywood Building Material (i.e., Veneer Sheets)

#### 2.2.1. Tensile Strength 

Tensile strength in longitudinal (L) direction was determined in accordance with the SIST EN 408:2010 standard [38]. The test specimens were made of single layers of veneer with dimensions 200 mm × 30 mm × 1.5 mm (L × T × R). The tensile strength in tangential direction was determined according to the ASTM D143:2000 [39] standard, where the test specimens with dimensions 60 mm × 60 mm × 15.6 mm and a width of 25 mm in the narrower part of the cross section were cut from 11E panel. The specimens were clamped in the jaws of the Zwick Z005 universal testing machine (Zwick Roell Group, Ulm, Germany) and tensioned at a rate of 0.3 mm/min. The tensile strength was then calculated using the following equation

(7) $$\sigma_{t}=\frac{F_{t\;max}}{b\;h}$$

where *F*_tmax_ is the failure force and *b* and *h* are the width and thickness of the specimens, respectively. The tensile strength in radial direction was not determined experimentally because there are no fractures in the radial direction in the fabricated flexural specimens as plane stress state in the TL plane is expected. Therefore, the same value was used for the radial tensile strength as for the tangential one.

#### 2.2.2. Compressive Strength

The compressive strength in the longitudinal (L), tangential (T) and radial (R) directions was determined on specimens measuring 15.6 mm × 15.6 mm × 15.6 mm which were cut from 11-ply boards (11E). Three specimens were loaded in the longitudinal direction, 3 in the tangential direction and 3 in the radial direction where the loading rate was 0.3 mm/min. The EN408:2010 [38] standard was used to determine compressive strength with the following equation

(8) $$\sigma_{c}=\frac{F_{c,prop}}{b\;l}$$

where *F*_c,prop_, is the failure force determined as the intersection between the loading curve and the parallel of the trend line from the initial linear part between 10% and 40% of the failure force with an offset of 0.01 × *h*, and *b* and *l* are the width and length of the specimen, respectively.

#### 2.2.3. Shear Strength

The shear strength *τ*_TL_ was determined by the asymmetric four-point bending (AFPB) test [40]. Specimens measuring 150 mm × 50 mm × 15.6 mm (T × L × R) were made from 11E plywood. The specimens were cut in the middle so that the cross-sectional height was 15.7 mm and the loading rate was 2 mm/min. The shear strength was calculated using equation [40]

(9) $$\tau_{TL}=\frac{P}{2bh}$$

where *P* is the force at which shear failure occurs, *b* is the cross-sectional height, which was 15.7 mm, and *h* is the cross-sectional thickness with the value of 15.6 mm.

The shear strengths in the RL and TR directions were calculated using Equation (10) [41], as well as the shear strength in the TL direction and compared with the experimentally determined values

(10) $$\tau_{RL}=\frac{1}{3}\;{\left[3\;\sigma_{uR}\sigma_{uL}\right]}^{0.5},\;\;\tau_{TL}=\frac{1}{3}\;{\left[3\;\sigma_{uT}\sigma_{uL}\right]}^{0.5}\;\tau_{RT}={\left[\frac{16}{{\left(K-1\right)}^{2}\;{\left(\sigma_{uR}+\sigma_{uT}\right)}^{2}}+\frac{1}{\sigma_{uR}\sigma_{uT}}-\frac{1}{\sigma_{uR}{}^{2}}-\frac{1}{\sigma_{uT}{}^{2}}\right]}^{-0.5}$$

where *σ*_uL_, *σ*_uR_ and *σ*_uT_ are the strengths in longitudinal, radial and tangential directions, respectively, and *K* is a constant which equals to 0.2 for hardwood.

#### 2.2.4. Modulus of Elasticity 

The modulus of elasticity was determined on 11-layer specimens cut from 11E plates. Three specimens with dimensions 410 mm × 40 mm × 15.6 mm in L × T × R and three specimens in T × L × R direction were cut from the plate to determine the elastic modulus in longitudinal and tangential directions, respectively. The specimens were loaded to four-point bending according to the EN408:2010 [38] standard, where the loading rate was 2.7 mm/min. The specimens were loaded to failure and then the modulus of elasticity was determined in the linear range between 0.2 and 0.3 *F*_max_ using the following equation:

(11) $$E_{L,T}=\frac{3al^{2}-4a^{3}}{4bh^{3}\left(\frac{w_{2}-w_{1}}{F_{2}-F_{1}}\right)}$$

where *F*_1_ and *F*_2_ are the forces at displacement *w*_1_ and *w*_2_, respectively, *b* and *h* are the width and thickness of the specimen, *l* is the distance between supports, which was 276 mm, and *a* is the distance between the support and the location of loading, which was 90 mm.

The modulus of elasticity in the radial direction was taken from literature [1], as exact value was not necessary because the plane stress state in TL plane was predicted. 

#### 2.2.5. Shear Modulus

The shear modulus in the TL direction was determined using the plate twist method [19]. Three plates measuring 145 mm × 145 mm were made from plywood 11E. The plates were then diagonally supported as well as loaded at distance of 195 mm. The shear modulus was then calculated using the following equation:

(12) $$G_{TL}=\frac{3\;B^{2}\;s}{4h^{3}}\cdot \frac{P}{\delta }$$

where *P* and *δ* are the shear force and deflection, respectively, and *s* is the correction factor related to the position of the clamp or load with respect to the diagonal of the specimen, calculated using

(13) $$s=3\cdot {\left(\frac{S}{D}\right)}^{2}-2\cdot \left(\frac{S}{D}\right)-2\cdot {\left(1-\frac{S}{D}\right)}^{2}\cdot ln\left(1-\frac{S}{D}\right)$$

where *S* is the distance between supports or between loads and *D* is the diagonal of the specimen.

The shear moduli in RL and TR directions were calculated according to Bachtiar et. al. [41], as well as the TL shear modulus, and compared with the measured values:

(14) $$G_{RL}={\left(\frac{\nu_{RL}+1}{E_{L}}+\frac{\nu_{LR}+1}{E_{R}}\right)}^{-1},\;\;\;\;\;G_{TL}={\left(\frac{\nu_{TL}+1}{E_{L}}+\frac{\nu_{LT}+1}{E_{T}}\right)}^{-1}\;G_{Rt}={\left(\frac{\nu_{TR}-1}{E_{R}}+\frac{\nu_{RT}-1}{E_{T}}+\frac{8}{\left(1-K\right)\;\left(E_{R}+E_{T}\right)}\right)}^{-1}$$

### 2.3. Plywood Failure Bending Forces Determination

**Figure 2:**
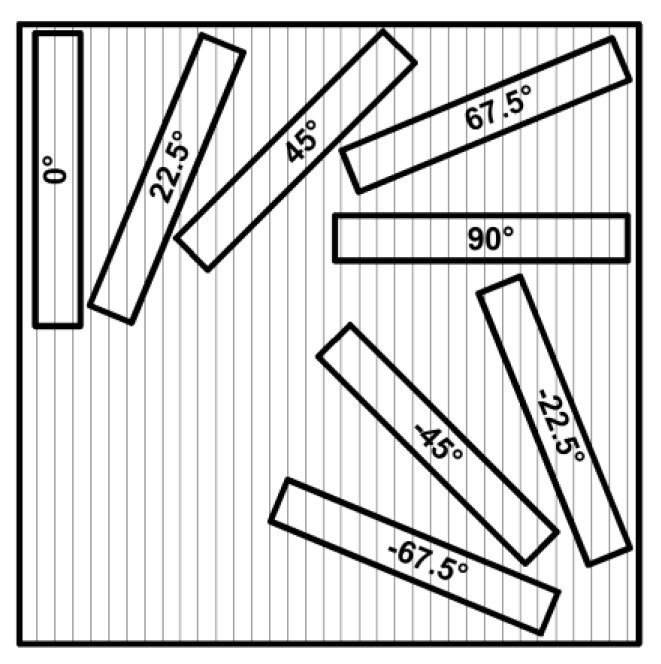
Scheme of cutting specimens from the boards. Numbers designate the first layer tissue orientation according to the individual specimen coordinate system as shown in Figure 1.

Specimens, 40 mm wide and 410 mm, 270 mm and 110 mm long, were prepared from 11-, 7- and 3-ply plates, respectively. 11A and 7A specimens were cut with an angular distribution of 22.5°: 0°, 22.5°, 45°, 67.5°, 90°, −22.5°, −45° and −67.5° (Figure 2), while the 11P, 7P and 3P specimens were cut only at the following angles due to symmetry: 0°, 22.5°, 45°, 67.5° and 90°. Since the boards were made from veneer with uniform mechanical properties, obtained from a log with uniform annual growth, only four specimens were made for each combination of tissue orientations. If there were major differences in mechanical properties between samples with the same combinations of tissue orientations, additional samples would be made.

The specimens were tested with four-point bending to the specimen failure. The distance between supports was 276 mm, 180 mm and 80 mm for the 11-, 7- and 3-ply plates, respectively, while the distance between loads and supports was 90 mm, 58 mm and 26 mm, respectively, and the loading rate was 2.7 mm/min.

The maximum force at which the specimen failed and the force at which the linear part of the curve changed to a nonlinear one was then determined from the measurements. The force at which the transition from the linear to the nonlinear part occurred was used as the failure force for the two reasons.

The first reason was that when wood specimens are subjected to bending loads, compression failure may occur first at the top of the specimen, but the specimen does not break in two. It is characteristic of wood that the compressive strengths are lower than the tensile one [1]. This means that when the wood is bent, the ultimate strength on the compressed top side is reached earlier than on the tensile bottom side. Although compressive failure occurs on the top side of the specimen, the specimen withstands the loads further where densification of wood tissue occurs. The compressive failure of the tissue can be identified from the loading curve as a transition from the linear to the nonlinear part, which is identical to the situation in the compression test. As the load increases, the tensile stresses at the bottom of the specimen further increase, where they eventually break when they reach the tensile strength or appropriate combination of normal and shear stresses.

The second reason for considering the transition force from the linear to the nonlinear part is that the tensile failure of the veneer in tangential direction may occur first on the bottom side of the specimen and only then compressive failure on the top side of the specimen. As shown by the results of the experimental work, veneer, unlike solid wood, has a lower tensile strength than compressive strength in tangential direction which is due to the way it is manufactured. Since the veneer in plywood is glued at different angles, local tensile failures in tangential direction are usually inhibited by the adjacent layer with more longitudinally oriented fibres, which also bear the load from the broken layer, which is not a major problem as the longitudinal strength is up to 30-times greater than the tangential transverse strength. Although the specimen has not yet collapsed on the lower tensile side, the undamaged cross-section of the specimen is smaller, which can be seen from the increase in the compliance of the specimen as it transitions from the linear to the nonlinear part of the loading curve.

The transition from the linear to the nonlinear part of the curve, and thus the determination of the failure force, was determined in a similar way to the compression test. From the linear part of the loading curve, a trend line was obtained from the measurements between 0.2 and 0.3 *F*_max_, and then a parallel was drawn representing the strain of 0.0002 mm/mm. The force of tissue failure was determined from the intersection between the offset line and the measurement curve.

### 2.4. Finite Element Modelling

**Figure 3:**
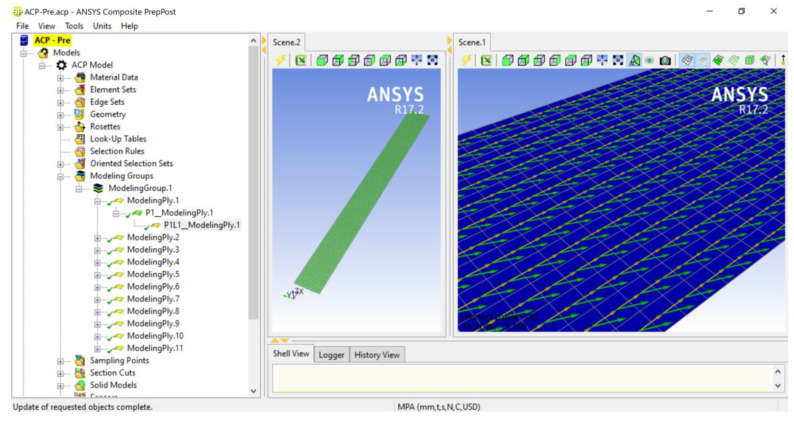
Composite finite element model.

All specimens tested experimentally for four-point bending with different fabric orientations and numbers of plies were also modelled using the finite element method with the ANSYS v17.2 (ANSYS, Inc., Canonsburg, PA, USA), Composite Module software (Figure 3). The size of the specimens and the spacing between supports and loads were the same as those in the experimental work. The specimens were modelled as planar elements with a thickness of 1.418 mm per layer and the size of elements of 3 mm.

Since a linear model was used, only the determination of the failure of the first most critical layer is credible. No further sequence of failure of other layers is possible with a linear model, as it does not include nonlinear tissue failure. The latter would only be possible with a non-linear model, which is beyond the scope of this study.

In the model, a force of 1000 N was applied to the specimen. Factors of safety (FOS) were calculated, defined as the ratio between the actual force and failure force where a safety factor of 1 means the failure of the layer. The safety factors were calculated for each layer using the max stress, Tsai-Wu, Puck, Tsai-Hill, Hoffmann and Hashin failure criteria (Table 1).

The failure criterion of Tsai-Wu (Table 1) includes a constant of *a*_xy_ that must be between −1 and 1, but despite numerous studies by different authors [32,42,43], there is still no universal equation for its determination. The following values were used in the study: −1, −0.7, −0.3, 0, 0.3, 0.6 and 1. For each value, the factors of safety and the corresponding failure force of the critical layer were calculated, and then compared with the measured force, where the coefficient of determination *R*^2^ between the calculated and the average of measured values was determined.

For the Puck criterion (Table 1), it is also necessary to determine the slopes $p_{∥⊥}^{\left(-\right)}$, $p_{∥⊥}^{\left(+\right)}$, $p_{⊥⊥}^{\left(-\right)}$ and $p_{⊥⊥}^{\left(+\right)}$ (in this paper designated as *p*) of the fracture curves. In the literature, there are only constants given for glass fibre/epoxy and carbon fibre/epoxy [25] between 0.2 and 0.3. Since the data for wood have not been found yet in the literature, the values 0.01, 0.15, 0.3 and 0.5 were considered in the study. For each individual value, the factor of safety and the failure force were calculated, and then compared with the measured values.

For the elastic modulus, shear moduli and strengths in the different directions, the values obtained from experimental work were used. The Poisson’s ratios were taken from the literature [1] where the values for *ν*_LT_, *ν*_LR_ and *ν*_TR_ were 0.518, 0.448 and 0.359, respectively.

Minimum safety factors for the weakest layer of the plate were determined using all the failure criteria, which were then multiplied by the load force. The calculated failure forces were then compared with the forces obtained from the experiment, so that for each criterion the coefficient of determination *R*^2^ between the calculated and the average of measured values was calculated.

### 2.5. Statistical Evaluation of Data

The average, standard deviation and coefficient of variation (COV) were calculated for the measured values of each group. The correlation between different groups of data (experimentally determined and theoretically calculated ones) was determined using the coefficient of determination (*R*^2^), defined as the proportion of variance in the first group of variables that is correlated with the second group of variables.

## 3. Results

### 3.1. Principle Mechanical Properties of Plywood Building Material (Veneer Sheets)

**Figure 4:**
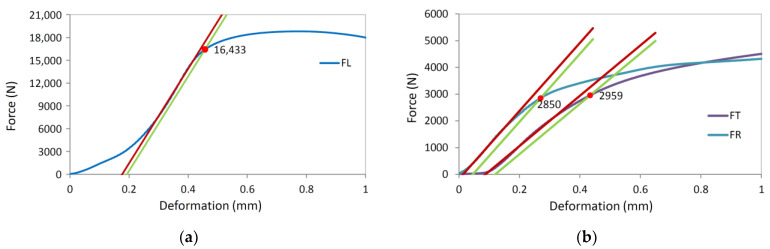
Measured forces and deformations in compression strength determination experiment. Red line represents linear trend between 10% and 40% of the failure force. Green line represents 1% strain offset of the red line: (**a**) Longitudinal direction; (**b**) Tangential and radial direction.

**Figure 5:**
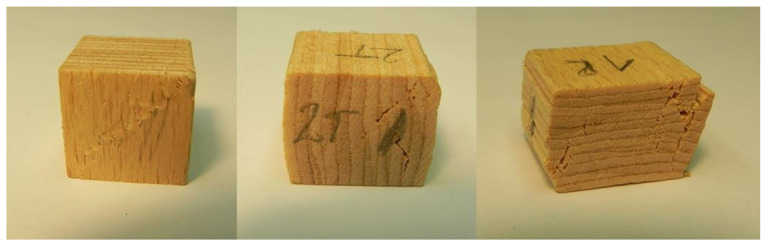
Samples for determining the pressure strength in longitudinal, tangential and radial direction.

Figure 4 shows the compressive strength measurements in longitudinal, tangential and radial directions along with the trend lines used to determine the failure force for the compressive strength calculation, while Figure 5 shows compressed specimens. In the initial phase of the measurement, the forces increase nonlinearly due to the adaptation of the specimen to the table of the testing machine. The nonlinear part is followed by a linear part and then again, a nonlinear part due to the local tissue failure. 

Table 3 shows the test results. The average compressive strengths in the longitudinal, tangential and radial directions are 65.4 MPa, 11.4 MPa and 11.1 MPa, respectively, while the average tensile strengths in the longitudinal and tangential directions are 96.8 MPa and 3.7 MPa, respectively. The tensile strength in the longitudinal direction is greater than the compressive strength and comparable to that in the literature [44,45] while the tensile strength in the tangential direction is much lower than the compressive one and also lower than stated in the literature which gives 9 MPa for beech in the tangential direction [1]. The reason for the lower tensile strength are microcracks that form when the veneer is peeled, and due to the microcracks, stress concentrations occur at tensile load. For the tensile strength in radial direction, the same value as in tangential direction was used as the plane stress state in TL plane is considered.

The average shear strength in the TL direction determined from the asymmetric four-point bending test is 9.3 MPa, which is higher than the literature data for LVL beech specimens [46,47]. The shear strength in the TL direction was also calculated using Equation (10), where the results was 10.9 MPa. Since the difference was not great a reasonable applicability of Equation (10) can be confirmed and further used to calculate shear strength in the TR direction where the result was 1.6 MPa, while the shear strength in RL direction was taken the same as in TL direction. 

The results for the modulus of elasticity and shear modulus are shown in Table 4. The average value of modulus of elasticity in longitudinal and tangential directions is 14,854 MPa and 984 MPa, respectively, and is comparable with the literature [44,45,46], while the modulus of elasticity in radial direction was taken from the literature [1]. The average value of the shear modulus in TL direction is 593 MPa, while the theoretically calculated value according to Equation (14) is 619 MPa, which corresponds to a difference of 4.2%. Since the difference was negligible, the applicability of Equation (14) was confirmed, and it was used to calculate the moduli in RL and RT directions, which were 1464 MPa and 388 MPa, respectively. The standard deviations of the measurements as well as the coefficient of variation were very small, which can be attributed to the relatively homogeneous specimens.

### 3.2. Failure of Four-Point Plywood Bending Specimens

**Figure 6:**
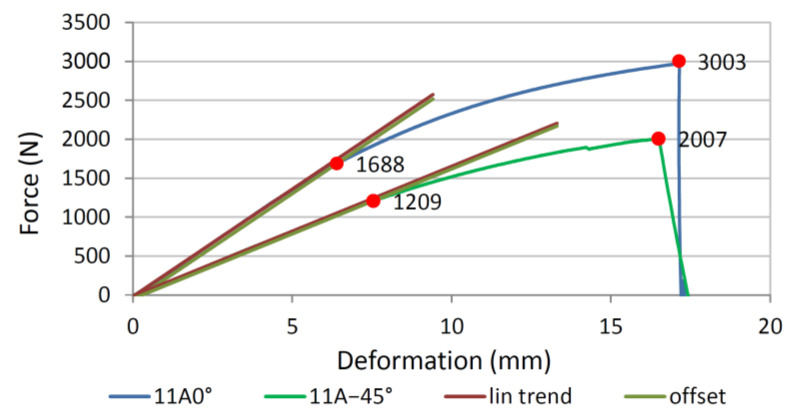
Forces and deformations of the four-point bending specimen 11A0° and 11A−45°.

**Figure 7:**
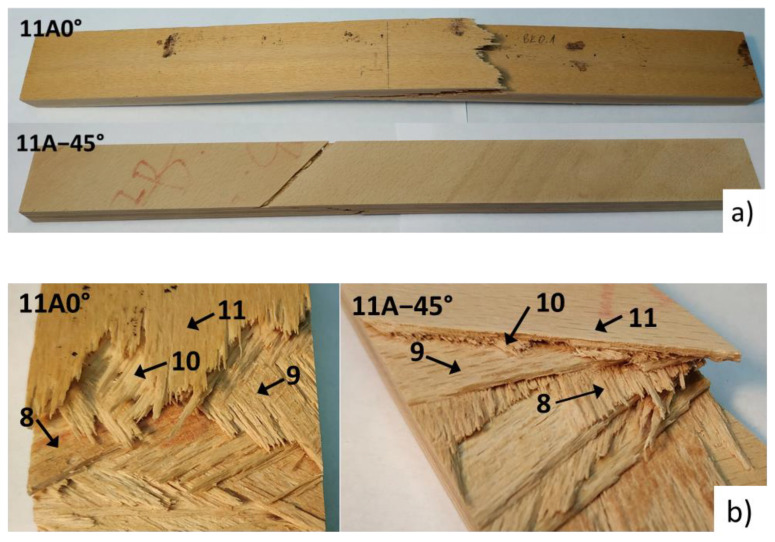
4-point bending specimens 11A0° and 11A−45°: (**a**) Whole specimens; (**b**) detail of the specimen underside with layer numbering.

Figure 6 shows the forces and deformations of the four-point bending loaded 11-layer specimen cut from plate 11A and with the direction of the first layer of 0° and −45°, while Figure 7 shows the corresponding specimens. The transition from the linear to the nonlinear part occurs for specimens 11A0° and 11A−45° at failure forces of 1688 N and 1209 N, while the final breaking force is 3003 and 2007 N, respectively.

**Figure 8:**
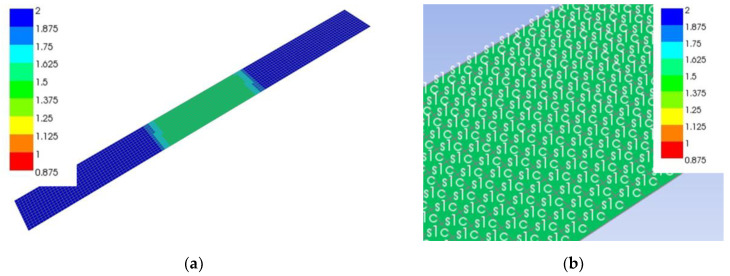
Distribution of safety factor (FOS) of layer No. 1 determined by the max stress criterion for specimen 11A0° loaded with a force of 1000 N: (**a**) Whole specimen; (**b**) detail of the middle part.

Table 5 and Table 6 show the min-max mid-sample FOS values for each layer for samples 11A0° and 11A−45°, respectively, for different failure criteria along with the failure mode according to the Puck, max stress and Hashin criteria, where the Tsai-Wu criterion has a constant *a*_xy_ equal to 1 and the Puck criterion has a constant *p* equal to 0.01. In Table 5, the sample 11A0° has the weakest upper layer No. 1, as it has the lowest min FOS value for all criteria and ranges from 1.33 to 1.54 for different criteria. The distribution of the FOS values together with failure mode is shown in Figure 8. For the first layer, all three criteria determined the same failure mode of compression failure in the fibre direction, which was expected since the angle of the fibres was 0°. The next weakest layer was the lowest layer No. 11, with the same fibre angle of 0°, but with tensile stresses. Again, all three criteria predict failure in the fibre direction, which is confirmed by Figure 7b, which nicely shows the failure of the lowest layer No. 11 in the fibre direction. This is to be expected, as compressive strength in longitudinal direction is lower than the tensile one. According to the measurement, the compressive failure of the top layer No. 1 occurred at the force of 1688 N, where the force of 1540 N was calculated by the Puck criteria, but the specimen did not break into two pieces as the top layer can still bear the compressive load despite the local compressive failure. By increasing the force and deformation, the failure of the bottom layer No. 11 followed at a measured force of 3003 N. According to the Puck criterion, the failure force of the lower layer is expected to be 2300 N, and according to the Tsai-Wu failure criterion, it is expected to be 2910 N. The theoretical forces are valid only under the assumption that the stress increases linearly with the force, which is not true in our case because the compressive stress in the upper layer does not increase with increasing force due to the failure of the tissue, and as such the calculated failure force for the lower layer No. 11 cannot be considered as completely valid. In the event that we wish to determine the actual failure force of the specimen at which the bottom layer would also fail, a nonlinear finite element model would be required, which is beyond the scope of the current research.

**Figure 9:**
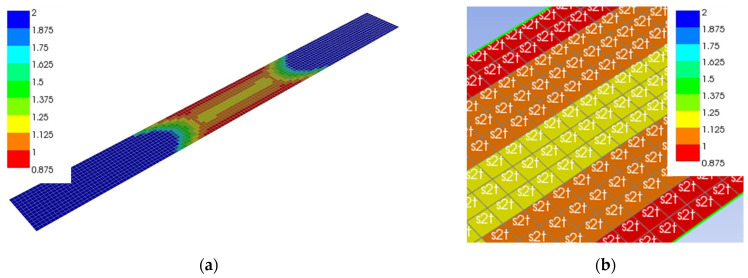
Distribution of the safety factor (FOS) of the layer No. 11 determined by the max stress criterion for specimen 11A−45° loaded with a force of 1000 N: (**a**) Whole specimen; (**b**) detail of the middle part.

The situation is opposite for sample 11A−45°, whose min-max mid-sample FOS are listed in Table 6. Here, layer No. 11 is the weakest, having the lowest min FOS for various criteria ranging from 0.8 to 0.87. Unlike sample 11A0°, where layer No. 1 had a uniform FOS distribution across the width, the FOS distribution in layer No. 11 of sample 11A−45° varied considerably from 0.87 to 1.13 for the max stress criterion as shown in Figure 9. The transverse tensile failure of the lower layer No. 11 occurs first, followed by layer No. 9, and only then occurs compressive failure at the top part of the specimen, as the tensile and compressive strengths in tangential direction were 3.7 MPa and 11.4 MPa, respectively. 

All the criteria predicted the failure of layers No. 11 and 9 in the transverse or shear direction, which can also be seen in Figure 7b. Puck’s criterion predicted failure under the combination of normal and shear stress (mode A), while the max stress and Hashin’s criterion predicted matrix failure due to transverse tension. According to Puck’s criterion, the lowest layer No. 11 would fail at a force of 820 N, whereas a force of 1209 N was determined in the experiment. The reason for the difference could be that despite the failure of layer No. 11 in the transverse direction, the stiffness drop is not affected because the load carrying capacity of layer No. 11 in the transverse direction is much lower than the load carrying capacity of layer No. 10, which transmits the load in the longitudinal direction of the failing layer No. 11.

**Figure 10:**
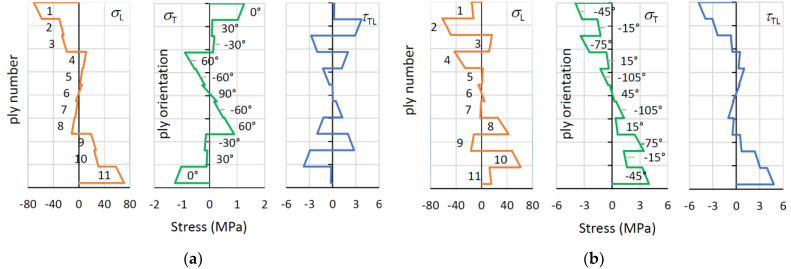
Longitudinal, tangential and shear stress distribution: (**a**) Specimen 11A0°, loading force 1688 N; (**b**) specimen 11A−45°, loading force 1209 N.

The cross-section distribution of longitudinal and tangential stresses for sample 11A0° at a load of 1688 N is shown in Figure 10a. For all layers, the longitudinal stress predominates, while the maximum tangential stress is 1.5 MPa, much lower than the tensile and compressive strengths, which are 3.7 MPa and 11.4 MPa, respectively. In addition, the maximum shear stresses do not exceed 4 MPa, which is again much lower than the shear strength of 9.3 MPa. Due to the predominant longitudinal stresses, all the outer layers break in the direction of the fibres, which is also predicted by all three failure criteria.

The situation is different for the sample of 11A−45 ° shown in Figure 10b for the loading force of 1209 N. The stresses in the outer layers are larger in tangential direction and are −4.6 and 4.6 MPa on the compression and tension sides of the specimen, respectively, which is more than the tensile strength of −3.7 MPa and implies to the failure of the corresponding layer. 

**Figure 11:**
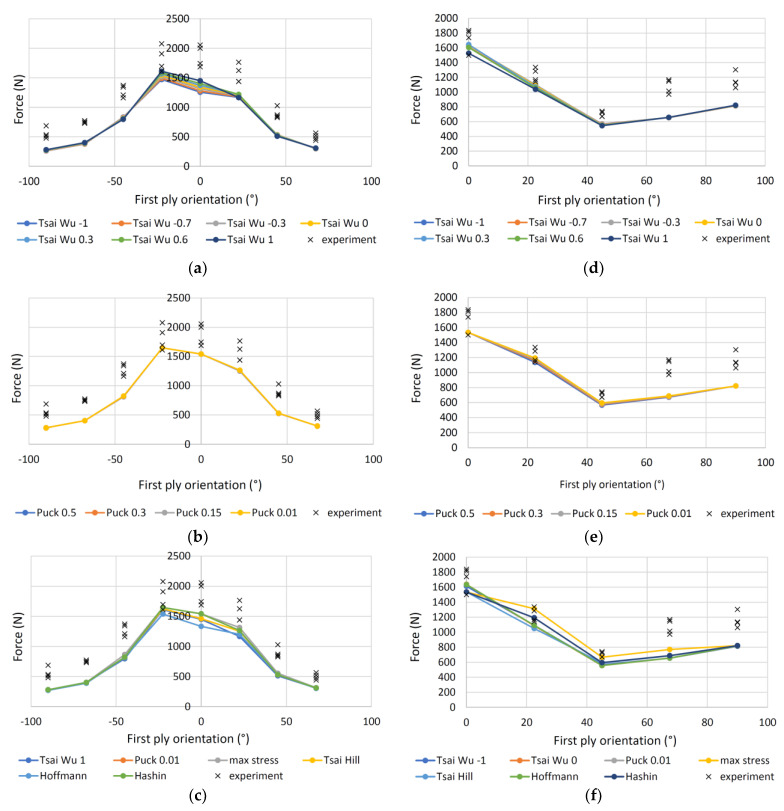
Measured and calculated forces of the four-point bending loaded 11-ply specimens: (**a**) Specimens 11A, Tsai-Wu criterion; (**b**) specimens 11A, Puck criterion; (**c**) specimens 11A, various criteria; (**d**) specimens 11P, Tsai-Wu criterion; (**e**) specimens 11P, Puck criterion; (**f**) specimens 11P, various criteria.

Figure 11 shows the experimentally determined and theoretically calculated forces of the four-point bending loaded 11-ply specimens. The measured forces vary a little and show a clear trend related to the different orientations of layers. The maximum load capacity for 11A specimen is at 0° and −22° for first ply orientation angle and then the forces decrease. Figure 11a,b shows the theoretically calculated failure forces according to the criteria of Tsai-Wu and Puck, respectively, based on a minimum factor of safety over the entire specimen cross-section for different values of the constants *a*_xy_ and *p*. The differences between individual values calculated with Tsai-Wu are not large, but they agree best with the criterion with constant *a*_xy_ = 1. The latter can also be seen in Table 7, where the coefficient of determination *R^2^* between the calculated values and the average of the measured forces is 0.551. In addition, for the Puck calculations, the differences are minimal for different constant values of *p*, where *R*^2^ for *p* = 0.01 is 0.636. 

The results for 11P samples are shown in Figure 11d-f. The Tsai-Wu criterion (Figure 11d) predicts the failure values well at smaller orientations, while at higher angles the deviation between the predicted and measured values is larger. The best prediction is at *a*_xy_ = 0, where *R*^2^ is 0.367. Even using the Puck criterion (Figure 11e), the forces differ only slightly at different *p* values, and the *R*^2^ is highest at 0.421 for a value of *p* = 0.01.

Figure 11c,f shows the results of all criteria for 11A and 11P samples, respectively. The max stress failure criterion has the best correlation with the highest *R*^2^, which is 0.674 for 11A samples and 0.538 for 11P samples. Max stress is followed by Hashin and Puck criteria in both groups. For the 11A samples, the difference is not very large, while it is larger for the 11P samples. For the 11A samples, then the criteria of Tsai Hill, Tsai Wu and Hoffman are followed, while for the 11P samples, the criteria of Hoffman, Tsai Wu and Tsai Hill are followed.

**Figure 12:**
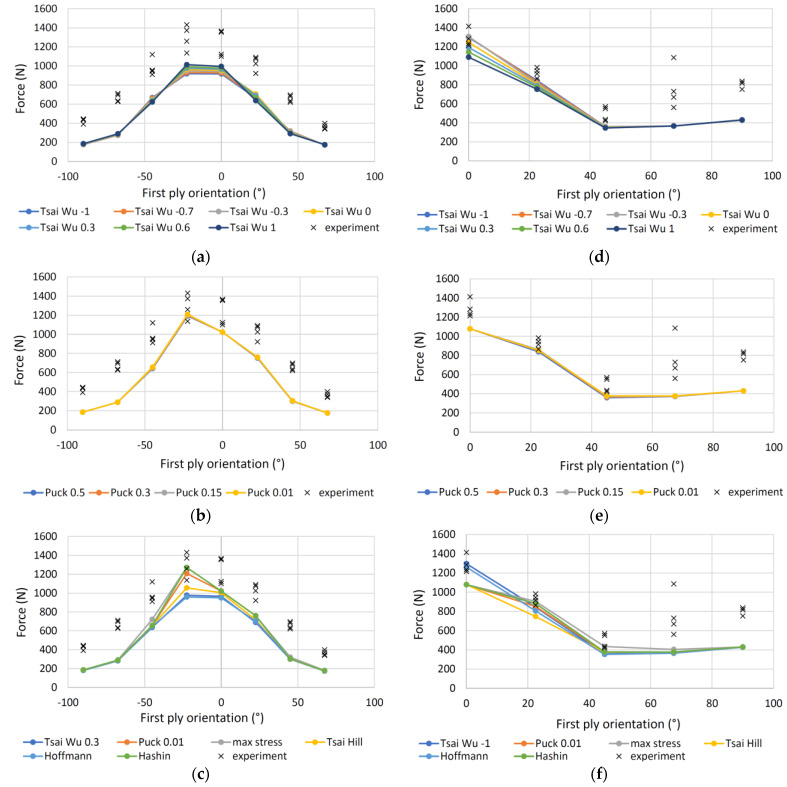
Measured and calculated forces of the 4-point bending loaded 7-ply specimens: (**a**) Specimens 7A, Tsai-Wu criterion; (**b**) specimens 7A, Puck criterion; (**c**) specimens 7A, various criteria; (**d**) specimens 7P, Tsai-Wu criterion; (**e**) specimens 7P, Puck criterion; (**f**) specimens 7P, various criteria.

The results for seven ply samples are shown in Figure 12. For 7A specimens the forces calculated by using the Tsai-Wu criterion (Figure 12a) differ only slightly for different values of *a*_xy_, having the best *R*^2^ (Table 7) of 0.133 for an *a*_xy_ = 0.3, while the forces calculated with the Puck criterion (Figure 12b) practically do not differ from each other and the highest coefficient of determination of 0.338 has a criterion with *p* = 0.01, as was the case for the 11-layer samples. The calculations for 7P samples are shown in Figure 12d,e. As with the 11P samples, Tsai-Wu (Figure 12d) predicts the forces well at smaller angles, while the deviation is larger at larger angles. The largest *R*^2^ has a criterion with *a*_xy_ = −1, while Puck’s criterion (Figure 12e) again has the largest *R*^2^ at a value of *p* = 0.01.

Figure 12c,f shows the results of all criteria for 7A and 7P samples, respectively. As with the 11-layer plates, the max stress criterion has the best *R*^2^, followed by Hashin, Puck, Tsai-Hill, Tsai-Wu and Hoffman for the 7A specimens, and Tsai-Wu, Hoffmann, Hashin and Tsai-Hill for the 7P specimens.

**Figure 13:**
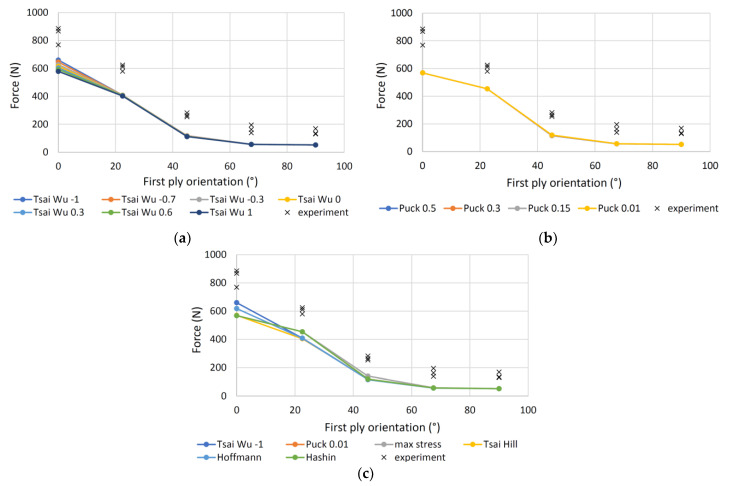
Measured and calculated forces of the four-point bending loaded 3P specimens: (**a**) Tsai-Wu criterion; (**b**) Puck criterion; (**c**) various failure criteria.

Figure 13 shows the results for the 3-layer samples. Tsai-Wu criterion (Figure 13a) has slightly larger deviations even at smaller angles and the best *R*^2^ at *a* = −1, similar to the 7P samples, while the Puck criterion (Figure 13b) has the largest *R*^2^ at *p* = 0.01. Comparison of all criteria is shown in Figure 13c. Tsai-Wu and Hoffmann criteria have a slightly better *R*^2^ than the max stress criterion, followed by the Hashin, Puck and Tsai-Hill failure criteria.

## 4. Discussion

Comparison of the coefficients of determination shows that all criteria agree better for the three- and 11-layer samples and worse for the seven-layer samples. For the 11- and seven-layer samples, the maximum stress criterion with the highest coefficient of determination has the best agreement between the calculated and measured values. In contrast, Tsai-Wu for the three-layer samples has a slightly higher *R*^2^ than the maximum stress criterion, while for the 11-layer and seven-layer samples, the *R*^2^ for Tsai-Wu is lower than most of the other criteria. It can be concluded from the study, that the maximum stress criterion is the most accurate criterion for predicting the failure criteria of wood-based materials such as plywood. The latter result is somewhat surprising since the maximum stress criterion is the simplest of all those studied and also does not take into account mutual interactions of normal stresses in different directions and the interaction of normal and shear stresses, which are essential for other unidirectional composites [25]. Hashin and Puck’s criteria also have high *R*^2^ values which, together with the maximum stress criterion, have similar conditions for failure of the tissue in the fibre direction, i.e., they compare only longitudinal stresses with corresponding strengths.

Similar results were obtained by others [22,48], who also investigated the application of the Tsai-Wu, Tsai-Hill, Hoffmann and maximum stress criteria to solid wood specimens. They found that the maximum stress and the Tsai-Hill criterion had the best correlation, followed by the Hoffman criterion, while the Tsai-Wu criterion had the worst correlation.

The study also found that the calculated values of Tsai-Wu criterion did not differ significantly at different values of *a*_xy_ in the entire range from −1 to 1. Slightly different results were obtained by others [21,49] for solid wood, where the agreements differed significantly at various *a*_xy_. Little effect on the results was also obtained for the *p* values of the Puck criterion, where there were practically no differences between the calculated values at different *p*.

Regardless of the type of criterion used, the theoretically calculated forces are smaller than the measured ones. One of the reasons could be that the failure criteria take into account the relationship between the actual stress and the strengths, with the material failing when stresses reach the strength. This is true for unidirectional composites and solid wood. The situation could be different for composites consisting of layers with various tissue orientations. Here, the adjacent layers prevent the layers from failing, especially when the fabric fails in the transverse direction, because the adjacent tissue, which has a more prolonged longitudinal tissue orientation, prevents the failure progression of the adjacent layer. 

However, in such cases, the studies performed may not provide an accurate prediction of tissue failure, especially if a linear model is used that predicts a linear increase in stresses with increasing force. In such cases it would be necessary to use a nonlinear model that takes into account the constraining effect of adjacent layers in the event of failure, as well as the limit stresses that certain layers should not exceed in the event of local failure.

The use of failure criteria, like any model, depends on the input data. However, if the correct input data is not precisely known, one may end up with inaccurate calculations, which is a particular problem with plywood. These are made up of individual layers of veneer, usually produced by peeling, and when veneer is produced there are large bending deformations in the tangential direction, resulting in local cracks. Because of the small cracks, the tensile strength in the tangential direction is much lower than the tensile strength of solid wood. For example, the tensile strength of solid wood in the tangential direction can be up to three-times higher than the tensile strength of peeled veneer. Therefore, it is important to consider real data in the modelling, which can be a problem if it is not available, and that the values of solid wood from the literature are taken into account.

## 5. Conclusions

The use of failure criteria is an important tool for combined loading that can be used to determine the force at which failure of the specimen occurs. These are important for composites as well as for solid wood, which is a distinctly orthotropic material with different strengths in different directions as well as different strengths depending on the sign of the load. In most cases, it is important to consider mutual interactions, which are taken into account by more demanding criteria that are essential for UD composites made of man-made materials. However, upon investigation on plywood specimens, the max stress criterion was found to have the best correlation between the calculated and measured values, followed by the Puck and Hashin criteria. A similar finding was made by some other authors for wood. 

Regardless of the criterion, the calculated values for plywood are smaller than those determined experimentally, which ultimately means that we are on the safe side.

The method presented for applying failure criteria to plywood proved to be a suitable novelty, but it should be emphasized that knowledge of the exact mechanical properties of the material from which the plywood is made is essential for the accuracy of the failure force determination.

By using the failure criteria in combination with the finite element method, both of which are based on a linear model, a failure force is obtained at which the weakest layer fails. For plywood made of wood with a lower compressive strength than tensile one, this usually means that in the flexural test the compressive failure occurs first on the upper compression side of the specimen and only later on the lower tensile side of the specimen. Despite the initial compression failure, the specimen does not break in two but continues to resist the increase in force. If it is necessary to obtain a force that breaks the specimen in two, a nonlinear model must be used.

## Figures and Tables

**Table 1:** Failure criteria. *σ*_x_ and *σ*_y_—normal stresses in x and y directions; *τ*_xy_—shear stress; *X* and *Y*—normal strengths (compressive or tensile) in the x and y directions; S—shear strength; *X*_t_ and *X*_c_—normal tensile and compressive strength in x direction; *Y*_t_ and *Y*_c_—normal tensile and compressive strength in y direction; *a*_xy_—interaction coefficient; . $R_{⊥}^{\left(+\right)}$ and $R_{⊥}^{\left(-\right)}$—normal tensile and compressive strength perpendicular to the fibres; $R_{⊥∥}$—shear strength; $p_{∥⊥}^{\left(-\right)}$, $p_{∥⊥}^{\left(+\right)}$, $p_{⊥⊥}^{\left(-\right)}$ and $p_{⊥⊥}^{\left(+\right)}$—slopes of the failure curves.

max stress [20]	$\left(\left|\frac{\sigma_{x}}{X}\right|,\left|\frac{\sigma_{y}}{Y}\right|,\left|\frac{\tau_{xy}}{S}\right|\right)=1$	(1)
Tsai-Hill [21]	${\left(\frac{\sigma_{x}}{X}\right)}^{2}-\frac{\sigma_{x}\sigma_{y}}{X^{2}}+{\left(\frac{\sigma_{y}}{Y}\right)}^{2}+{\left(\frac{\tau_{xy}}{S}\right)}^{2}=1$	(2)
Hoffman [22]	$\frac{\sigma_{x}{}^{2}}{X_{t}X_{c}}+\frac{\sigma_{y}{}^{2}}{Y_{t}Y_{c}}+\frac{\tau_{xy}{}^{2}}{S^{2}}-\frac{\sigma_{x}\sigma_{y}}{X_{t}X_{c}}+\frac{\sigma_{x}}{X_{t}X_{c}}+\frac{\sigma_{y}}{Y_{t}Y_{c}}=1$	(3)
Hashin [22]	${\left(\frac{\sigma_{x}}{X_{t}}\right)}^{2}+{\left(\frac{\tau_{xy}}{S}\right)}^{2}=1,\sigma_{x}>0;-\frac{\sigma_{x}}{X_{c}}=1,\sigma_{x}<0$ ${\left(\frac{\sigma_{y}}{Y_{t}}\right)}^{2}+{\left(\frac{\tau_{xy}}{S}\right)}^{2}=1,\sigma_{y}>0$ ${\left(\frac{\sigma_{y}}{2S}\right)}^{2}+{\left(\frac{\tau_{xy}}{S}\right)}^{2}+\left[{\left(\frac{Y_{c}}{2S}\right)}^{2}-1\right]\frac{\sigma_{y}}{Y_{c}}=1,\sigma_{y}<0$	(4)
Tsai-Wu [23]	$\left(\frac{1}{X_{t}}-\frac{1}{X_{c}}\right)\sigma_{x}+\left(\frac{1}{Y_{t}}-\frac{1}{Y_{c}}\right)\sigma_{y}+\frac{1}{X_{t}X_{c}}\sigma_{x}^{2}+\frac{1}{Y_{t}Y_{c}}\sigma_{y}^{2}+2a_{xy}\sqrt{\left(\frac{1}{X_{t}X_{c}}\frac{1}{Y_{t}Y_{c}}\sigma_{x}\sigma_{y}\right)}+\frac{1}{S^{2}}\tau_{xy}^{2}=1$	(5)
Puck [24,25]	$\left|\frac{\sigma_{x}}{X_{t}}\right|=1,\sigma_{x}>0;\left|\frac{\sigma_{x}}{X_{c}}\right|=1,\sigma_{x}<0$ $\sqrt{{\left(\frac{\tau_{xy}}{R_{⊥∥}}\right)}^{2}+{\left(1-p_{⊥∥}^{\left(+\right)}\frac{R_{⊥}^{\left(+\right)}}{R_{⊥∥}}\right)}^{2}{\left(\frac{\sigma_{y}}{R_{⊥}^{\left(+\right)}}\right)}^{2}}+p_{⊥∥}^{\left(+\right)}\frac{\sigma_{y}}{R_{⊥∥}}=1,\sigma_{y}\geq 0$ $\frac{1}{R_{⊥∥}}\left(\sqrt{\;\tau_{xy}^{2}+{\left(p_{∥⊥}^{\left(-\right)}\sigma_{y}\right)}^{2}}+p_{∥⊥}^{\left(-\right)}\sigma_{y}\right)=1,\sigma_{y}<0,\left|\frac{\sigma_{y}}{\tau_{xy}}\right|\leq \frac{R_{⊥⊥}^{A}}{\left|\tau_{xyc}\right|}$ $\left[{\left(\frac{\tau_{xy}}{2\left(1+p_{⊥⊥}^{\left(-\right)}\right)R_{⊥∥}}\right)}^{2}+{\left(\frac{\sigma_{y}}{R_{⊥}^{\left(-\right)}}\right)}^{2}\right]\frac{R_{⊥}^{\left(-\right)}}{-\sigma_{y}}=1,\sigma_{y}<0,\left|\frac{\sigma_{y}}{\tau_{xy}}\right|\geq \frac{R_{⊥⊥}^{A}}{\left|\tau_{xyc}\right|}$	(6)

**Table 2:** Tissue orientations (*θ*) of individual layers for 3-, 7- and 11-layer beech veneer plywood.

Ply no.	11 Layers			7 Layers		3 Layers
	11E (°)	11A (°)	11P (°)	7A (°)	7P (°)	3P (°)
1	0	0	0	0	0	0
2	0	30	90	45	90	90
3	0	−30	0	−45	0	0
4	0	60	90	90	90	-
5	0	−60	0	−45	0	-
6	0	90	90	45	90	-
7	0	−60	0	0	0	-
8	0	60	90	-	-	-
9	0	−30	0	-	-	-
10	0	30	90	-	-	-
11	0	0	0	-	-	-

**Table 3:** Experimentally determined normal and shear strength in longitudinal (L), tangential (T) and radial (R) direction.

Specimen	Compression (MPa)			Tension (MPa)			Shear (MPa)			
	L	T	R	L	T	R	TL	TL [41]	RL	TR
1	66.9	11.5	11.5	89.9	3.9	-	9.2	-	-	-
2	63.6	11.3	11.0	115.1	3.8	-	9.9	-	-	-
3	65.6	11.4	10.9	95.3	3.4	-	8.7	-	-	-
4	-	-	-	94.2	3.7	-	-	-	-	-
5	-	-	-	99.2	-	-	-	-	-	-
6	-	-	-	83.1	-	-	-	-	-	-
7	-	-	-	100.7	-	-	-	-	-	-
**Avg**	65.4	11.4	11.1	96.8	3.7	3.7	9.3	10.9	9.3	1.6
**Std. dev.**	1.7	0.1	0.3	10.0	0.2	-	0.6	-	-	-
**COV (%)**	2.6	0.9	2.6	10.3	5.7	-	6.7	-	-	-

**Table 4:** Modulus of elasticity and shear modulus (in MPa).

Specimen	Experiment		Kollman [1]	Experiment	Equation 14		
	*E*_L_	*E*_T_	*E*_R_	*G*_TL_	*G*_TL_	*G*_RL_	*G*_RT_
1	14,578	991	-	586	-	-	-
2	14,597	992	-	590	-	-	-
3	14,578	968	-	604	-	-	-
**Avg**	14,584	984	2380	593	619	1464	388
**St. dev.**	11	14	-	9	-	-	-
**COV (%)**	0.08	1.38	-	1.59	-	-	-

**Table 5:** Calculated factor of safety (FOS) at loading force of 1000 N for various failure criteria for sample 11A0°. (Failure modes: Puck: f—fibre failure, mA—inter-fibre failure in mode A, mB—inter-fibre failure in mode B; max stress: 1c and 1t—compression and tension failure in direction 1; 2c and 2t—compression and tension failure in direction 2; 12—shear failure; Hashin: f—fibre failure, m—matrix failure).

Ply no.	Ply Orientation (°)	Tsai-Wu		Tsai-Hill		Hoffman		Puck			Max Stress			Hashin		
		FOS		FOS		FOS		FOS		Failure	FOS		Failure	FOS		Failure
		Min	Max	Min	Max	Min	Max	Min	Max		Min	Max		Min	Max	
1	0	1.45	1.46	1.46	1.47	1.33	1.35	1.54	1.55	f	1.54	1.55	1c	1.54	1.55	f
2	30	2.61	2.69	2.71	2.79	2.58	2.69	3.14	3.24	mA	3.56	3.77	1c	3.56	3.77	f
3	−30	3.09	3.17	3.21	3.30	3.02	3.11	3.66	3.79	mA	3.88	4.10	1c	3.88	4.10	f
4	60	10.1	10.4	6.56	6.60	8.30	8.36	7.93	8.14	-	7.93	8.22	-	6.91	7.04	-
5	−60	15.9	16.2	11.0	11.2	13.9	14.1	12.4	12.7	-	12.4	12.6	-	11.0	11.2	-
6	90	16.3	16.7	15.8	16.1	13.9	14.1	18.7	19.1	-	22.3	22.9	-	22.3	22.9	-
7	−60	7.55	7.74	8.04	8.30	7.02	7.26	8.88	8.97	-	12.4	12.6	-	8.89	8.98	-
8	60	4.25	4.35	4.43	4.57	3.87	3.98	5.10	5.22	-	6.60	6.97	-	5.13	5.24	-
9	−30	4.59	4.63	4.10	4.15	4.44	4.50	4.80	4.87	-	5.66	5.81	-	4.11	4.16	-
10	30	3.52	3.58	3.29	3.34	3.52	3.53	3.86	3.91	mB	4.14	4.20	-	3.30	3.34	f
11	0	2.91	2.94	2.26	2.26	2.49	2.50	2.30	2.31	f	2.30	2.31	1t	2.30	2.31	f

**Table 6:** Calculated factor of safety (FOS) at loading force of 1000 N for various failure criteria for sample 11A−45°. (Failure modes: Puck: f—fibre failure, mA—inter-fibre failure in mode A, mB—inter-fibre failure in mode B; max stress: 1c and 1t—compression and tension failure in direction 1, 2c and 2t—compression and tension failure in direction 2, 12—shear failure; Hashin: f—fibre failure, m—matrix failure).

Ply No.	Ply Orientation (°)	Tsai-Wu		Tsai-Hill		Hoffman		Puck			Max Stress			Hashin		
		FOS		FOS		FOS		FOS		Failure	FOS		Failure	FOS		Failure
		Min	Max	Min	Max	Min	Max	Min	Max		Min	Max		Min	Max	
1	−45	1.93	2.01	1.84	1.89	2.11	2.18	2.12	2.25	mC	2.33	2.65	12	1.73	1.78	m
2	−15	1.22	1.27	1.21	1.27	1.37	1.51	1.27	1.41	f	1.27	1.41	1c	1.27	1.41	f
3	−75	4.44	5.28	3.21	3.36	3.44	3.74	3.54	3.97	mC	3.54	4.05	2c	3.53	3.88	m
4	15	1.71	1.87	1.68	1.85	1.89	2.02	1.68	1.85	f	1.68	1.85	1c	1.68	1.85	f
5	−105	6.79	9.64	6.56	7.58	7.51	8.83	7.71	8.70	-	9.01	10.7	-	6.21	7.15	-
6	45	9.44	11.1	10.6	12.8	10.9	12.7	11.5	14.6	-	11.5	16.7	-	11.5	14.9	-
7	−105	2.95	3.25	3.08	3.29	3.08	3.18	3.08	3.31	mA	3.28	3.47	2t	3.08	3.31	m
8	15	1.91	2.24	2.26	2.58	2.14	2.45	2.50	2.75	f	2.50	2.75	1t	2.50	2.73	f
9	−75	1.15	1.29	1.12	1.25	1.07	1.18	1.15	1.31	mA	1.15	1.32	2t	1.15	1.31	m
10	−15	1.06	1.25	1.24	1.46	1.23	1.41	1.44	1.68	mA	1.77	2.02	1t	1.53	1.72	f
11	−45	0.80	0.95	0.82	1.01	0.82	1.01	0.82	1.02	mA	0.87	1.13	2t	0.82	1.02	m

**Table 7:** The coefficient of determination (*R*^2^) for different failure criteria between the calculated and the average of measured values of the failure forces. The green colour indicates the criterion with the best correlation of all criteria, and the bold values the best correlation within each criterion.

	*a*_xy_ (Tsai-Wu)/*p* (Puck)	11A	11P	7A	7P	3P
Tsai Wu	−1	0.431	0.358	0.060	***0.024***	***0.696***
	−0.7	0.456	0.364	0.081	0.016	0.680
	−0.3	0.487	0.371	0.109	0.005	0.659
	0	0.508	***0.367***	0.132	−0.008	0.643
	0.3	0.530	0.362	***0.133***	−0.039	0.626
	0.6	0.547	0.339	0.128	−0.086	0.609
	1	***0.551***	0.282	0.122	−0.165	0.587
Puck	0.5	0.627	0.371	0.318	−0.087	0.625
	0.3	0.631	0.394	0.329	−0.067	0.627
	0.15	0.634	0.409	0.334	−0.057	0.627
	0.01	***0.636***	***0.421***	***0.338***	***−0.053***	***0.628***
max stress		0.674	0.538	0.405	0.052	0.644
Tsai Hill		0.600	0.361	0.242	−0.133	0.585
Hoffmann		0.503	0.369	0.126	−0.002	0.648
Hashin		0.636	0.422	0.347	−0.042	0.628

## Data Availability

Not applicable.
